# Complete Genome Sequence of *Rehmannia Mosaic Virus* Infecting *Rehmannia glutinosa* in South Korea

**DOI:** 10.1128/genomeA.01595-15

**Published:** 2016-01-28

**Authors:** Seungmo Lim, Fumei Zhao, Ran Hee Yoo, Davaajargal Igori, Jae Cheol Jeong, Haeng-Soon Lee, Sang-Soo Kwak, Jae Sun Moon

**Affiliations:** a Plant Systems Engineering Research Center, Korea Research Institute of Bioscience and Biotechnology, Daejeon, Republic of Korea; b Biosystems and Bioengineering Program, University of Science and Technology, Daejeon, Republic of Korea

## Abstract

The complete genome sequence of a South Korean isolate of *Rehmannia mosaic virus* (ReMV) infecting *Rehmannia glutinosa* was determined through next-generation sequencing and Sanger sequencing. To our knowledge, this is the first report of a natural infection of *R. glutinosa* by ReMV in South Korea.

## GENOME ANNOUNCEMENT

*Rehmannia mosaic virus* (ReMV), a member of the genus *Tobamovirus* in the family *Virgaviridae*, was first discovered from *Rehmannia glutinosa* in China (1). The medicinal plant *R. glutinosa* is commonly known for its beneficial pharmacological effects on the human body (2). To date, the complete genomic sequences of two ReMV isolates from *Rehmannia* plants have been determined in China, and interestingly, another complete genomic sequence of ReMV was identified from chili pepper (*Capsicum annuum*) in Japan (1, 3).

In May 2014, one *R. glutinosa* sample showing symptoms of leaf mottling and yellowing was collected in Eumseong, South Korea. To identify the causal agent, the sample was identified using the Illumina HiSeq 2500 paired-end RNA sequencing and data analysis process, as described in a previous study (4). Consequently, a 6,377-nucleotide (nt)-long nearly complete genome sequence of ReMV was obtained from the *Rehmannia* sample. To confirm and determine complete genome sequence of the identified ReMV, primers were designed based on the sequence of the contig. Total RNA was extracted from the symptomatic leaves using TRI reagent (Molecular Research Center, Cincinnati, OH, USA), and cDNA was synthesized using a random N25 primer and RevertAid reverse transcriptase (Thermo Scientific, Waltham, MA, USA). PCR products covering the entire virus genome except for both ends were amplified with AccuPower ProFi Taq PCR PreMix (Bioneer, Daejeon, South Korea) and then cloned into the RBC T&A cloning vector (RBC Bioscience, Taipei, Taiwan) for sequencing by Macrogen (Daejeon, South Korea). Both ends of the genome were determined by rapid amplification of cDNA ends (RACEs) using the 5′/3′ RACE system (Invitrogen, Carlsbad, CA, USA). Due to lack of a polyadenosine tail at the 3′ ends of the tobamovirus genomes, the total RNA was adenylated using a poly(A) polymerase tailing kit (Epicentre, Madison, WI, USA) for the 3′ RACE.

The complete genome sequence of ReMV isolate ES (GenBank accession no. KU133476) was composed of 6,395 nt, including 5′ and 3′ untranslated regions with 71 and 204 nt, respectively. The virus shared higher nucleotide sequence identities with ReMV isolates Henan and Shanxi sampled from *Rehmannia* plants (GenBank accession no. EF375551, 99% identity; and GenBank accession no. JX575184, 97% identity, respectively) than ReMV isolate Japanese sampled from *C. annuum* (GenBank accession no. AB628188, 94% identity). Four open reading frames (ORFs) of ReMV isolate ES were predicted by the DNAMAN 5.0 program (Lynnon Biosoft, Quebec, Canada). ReMV is supposed to produce ORF1-ORF2 fusion protein via readthrough of a UAG termination codon of ORF1 (1, 5). The highest amino acid sequence identities between the ES isolate and other ReMV isolates were 99% for ORF1 (replicase), 99% for ORF1-ORF2 fusion (replicase), 99% for ORF3 (movement protein), and 99% for ORF4 (coat protein).

In this work, we identified a South Korean isolate of ReMV using RNA sequencing of a *R. glutinosa* sample. To our knowledge, this is the first report of ReMV in South Korea.

### Nucleotide sequence accession number.

The complete genome sequence of *Rehmannia mosaic virus* isolate ES has been deposited in GenBank under the accession number KU133476.
